# Measurements of cervical lymph nodes in children on computed tomography

**DOI:** 10.1007/s00247-019-04595-y

**Published:** 2019-12-18

**Authors:** Suzanne Spijkers, Annemieke S. Littooij, Rutger A. J. Nievelstein

**Affiliations:** 1 Department of Radiology and Nuclear Medicine, University Medical Centre Utrecht/Wilhelmina Children’s Hospital, Heidelberglaan 100, 3584 CX Utrecht, The Netherlands; 2 Department of Radiology and Nuclear Medicine, Princess Máxima Centre for Paediatric Oncology, Utrecht, The Netherlands

**Keywords:** Cervical spine, Child, Computed tomography, Diagnostic imaging, Lymph nodes, Lymphadenopathy, Normal values

## Abstract

**Background:**

No normal measurements or specific size criteria have been described for cervical lymph nodes in children.

**Objective:**

To determine the normal measurements of cervical lymph nodes in children on CT.

**Materials and methods:**

We included 142 children (ages 1–17 years) who underwent cervical CT examination after high-energy trauma. We evaluated axial and coronal 2-mm reconstructions for lymph nodes at six cervical levels. For the largest lymph node at each level, we measured diameters in both the long and short axial axes and the long coronal axis.

**Results:**

A total of 733 lymph nodes were measured in 142 children (62% boys, 38% girls). The greatest measured diameters were 14 mm for the short axis in the axial plane, 24 mm for the long axis in the axial plane and 28 mm for the long axis in the coronal plane. The Pearson correlation coefficient for age and lymph node size at Levels IV–VI was in the range of 0.19–0.47.

**Conclusion:**

Lymph nodes with an axial short-axis diameter exceeding 15 mm for Level II and 10 mm for all other cervical levels are uncommon in otherwise healthy children.

## Introduction

One of the possible symptoms of infectious or malignant disease in children is lymphadenopathy. Large lymph nodes are a common finding in otherwise healthy children [1, 2]. For correct diagnosis of enlarged cervical lymph nodes on ultrasound, CT or MRI, it is important that normal short- and long-axis diameters of lymph nodes are known for all cervical levels and for all ages.

Cervical lymphadenopathy in children is widely addressed in the literature, but this is often in terms of localisation and abnormal appearance [3]. No normal measurements or specific size criteria have been described for cervical lymphadenopathy in children and generally, the normal measurements as described for adults are applied for children as well. Lymph nodes with a short axis greater than 10 mm (mm) are usually considered abnormal; exceptions are deep cervical lymph nodes, in which a maximum diameter of 15 mm is considered within normal limits [1, 2, 4–7].

Although lymph nodes are typically measured in the axial plane, the growth and alignment of a lymph node might not correspond to this plane. Classification of lymphadenopathy was shown to differ among the axial, coronal and sagittal planes when applying size criteria based on the axial plane [8]. Axial measurements were found to be slightly smaller compared to measurements in the coronal and sagittal planes [8].

The body size changes continuously during childhood and the occurrence of large cervical lymph nodes is common in otherwise healthy children [1]. Reportedly, lymph nodes with a short axial axis greater than 10 mm are seen in up to 90% of children 4–8 years old [9]. Therefore, lymph node measurements are likely to differ over time and among ages, and applying adult standard values to children of all ages might not be sufficient for correct diagnosis of lymphadenopathy. For lymph nodes in the chest this influence of age on lymph node size was already shown [10].

Our aim was to analyse CT examinations in children after high-energy trauma to provide normal measurements of cervical lymph nodes for the paediatric population and to determine whether lymph node size changes by anatomical location and with the child’s age.

## Materials and methods

### Study population

We selected for this retrospective study all children aged 1–17 years who presented at the University Medical Centre Utrecht between January 2012 and July 2014 after high-energy trauma and underwent contrast-enhanced CT. We excluded CT examinations where no intravenous contrast medium was used and cases in which no cervical CT was available, as well as examinations in children diagnosed with malignancies that might affect lymph node size (e.g., lymphomas, leukaemia). Our institutional review board approved this study. For this retrospective study formal consent was not required.

### Computed tomography technique and image interpretation

All children received intravenous contrast medium (2 mL/kg body weight, in accordance with the local trauma protocol). The CT examinations were obtained with 16×0.75-mm collimation (Mx8000 IDT or Brilliance 16P), 64×0.625-mm collimation (Brilliance 64) or 128×0.625-mm collimation (Brilliance iCT) scanner, all from Philips Medical Systems (Cleveland, OH). Exposure settings (adjusted to patient size) ranged from 19 mAs to 307 mAs and 80 kV to 120 kV. Axial and coronal images were reconstructed from the frontal sinuses to the aortic arch. Thin-slice images were reconstructed with 0.8–1.0-mm thickness at 0.5–0.8-mm intervals and stored in a 512×512 data matrix.

One observer (S.S., with 2 years of experience) evaluated all CT examinations. Both the long and short axes in the axial plane and the long axis in the coronal plane of the largest lymph node at each level were measured. To calculate interobserver variability, a paediatric radiologist (A.S.L., with 10 years of experience) evaluated a subset of 20 CT examinations selected at random from all included CT examinations (with the premise of selecting at least one of each available age). The following lymph node levels were evaluated [11]:

- Level I: submental and submandibular lymph nodes, anterior to the posterior border of the submandibular glands;
- Level II: lymph nodes located posterior to the back of the submandibular glands and anterior to the back of the sternocleidomastoid muscle, between skull base and hyoid bone;
- Level III: lymph nodes located anterior to the posterior border of the sternocleidomastoid muscle and between hyoid bone and cricoid cartilage;
- Level IV: lymph nodes located between the cricoid cartilage and the clavicle;
- Level V: lymph nodes posterior to the sternocleidomastoid muscle and anterior to the trapezius muscle, between skull base and clavicle; and
- Level VI: prelaryngeal and pretracheal lymph nodes, from hyoid bone to the manubrium, anterior to Levels III and IV.

### Statistical analysis

Analyses of the data were performed using Statistical Package for the Social Sciences (SPSS) version 25.0 for Windows (IBM, Armonk, NY). Lymph node size was analysed per age and lymph node station. Median measurements, interquartile range (IQR) and maximum diameters were calculated for each age group. To identify whether adult guidelines for lymph node size are applicable in children, we calculated upper limits for normal ranges (mean value + 1.96 standard deviations [SD]) per level and axis using the logarithmic transformed data; we compared those measurements to the currently used adult guidelines for lymph node size (10-mm short-axial-axis upper limit, except for deep cervical nodes, in which an upper limit of 15 mm is considered normal) [1, 2, 4–7]. After performing a log transformation to normalise the data, we calculated Pearson correlation coefficients between age and lymph node size. Interobserver agreement for the CT examinations evaluated by both readers was assessed using a Bland–Altman plot.

## Results

We retrospectively identified 182 CT examinations after high-energy trauma in children; of these examinations, 142 CT scans were eligible for inclusion in this study. Forty children were excluded for the following reasons: cervical CT scan without intravenous contrast medium (*n*=10), presence of malignancies that might affect lymph node size (*n*=19), absence of a cervical CT scan (*n*=11). Thus, 142 children ages 1–17 years old were included (62% boys, 38% girls) and an overall total of 733 lymph nodes were measured. In nearly all children lymph nodes were found at Levels I–V (97%, 99%, 96%, 92% and 98%, respectively). At Level VI, lymph nodes were less prevalent (36%). Tables 1, 2 and 3 show median values, interquartile ranges (IQR) and maximum size diameters for the short-axis diameter in the axial plane (Table 1), the long-axis diameter in the axial plane (Table 2) and the long-axis diameter in the coronal plane (Table 3) of the largest lymph node at each level.

**Table 1:** Cervical lymph node short-axis size in millimetres in the axial plane by anatomical location

Age *(yr)*	Sub-jects (*n)*	Level I			Level II			Level III			Level IV			Level V			Level VI		
		*n* (%)	Median (IQR)	Max	*n* (%)	Median (IQR)	Max	*n* (%)	Median (IQR)	Max	*n* (%)	Median (IQR)	Max	*n* (%)	Median (IQR)	Max	*n* (%)	Median (IQR)	Max
1	6	6 (100)	6 (5.3–6.0)	7	6 (100)	6.5 (5.3–7.8)	8	6 (100)	5 (3.5–5.0)	6	3 (50)	3 (2.5–3.5)	4	6 (100)	4.5 (3.3–5.0)	6	2 (33)	2 (1.5–2.5)	3
2	7	7 (100)	5 (5.0–5.5)	6	7 (100)	8 (6.0–8.5)	9	6 (86)	4.5 (4.0–5.0)	6	7 (100)	3 (3.0–4.0)	4	7 (100)	4 (3.0–4.5)	6	1 (14)	2	2
3	6	6 (100)	5 (5.0–5.8)	6	6 (100)	7 (6.3–7.0)	11	6 (100)	4.5 (4.0–5.8)	6	5 (83)	4 (4.0–4.0)	4	6 (100)	4 (4.0–4.8)	5	3 (50)	3 (2.0–2.5)	3
4	3	3 (100)	5 (4.0–5.5)	6	3 (100)	7 (7.0–7.5)	8	3 (100)	4 (4.0–5.8)	7	3 (100)	3	3	3 (100)	4 (3.5–4.0)	4	1 (33)	3	3
5	5	5 (100)	6 (4.0–5.5)	7	5 (100)	9 (6.0–9.0)	9	5 (100)	5 (4.0–5.0)	6	5 (100)	4 (4.0–4.0)	5	5 (100)	4 (4.0–4.0)	4	0 (0)	–	–
6	6	5 (83)	5 (4.0–5.0)	5	5 (83)	8 (8.0–9.0)	12	5 (83)	4 (4.0–6.0)	6	5 (83)	5 (3.0–5.0)	5	6 (100)	3.5 (3.0–4.0)	5	2 (33)	3 (2.5–3.5)	4
7	4	4 (100)	6.5 (5.8–7.0)	7	4 (100)	6.5 (6.0–7.0)	7	4 (100)	4.5 (3.8–5.5)	7	4 (100)	3 (3.0–3.3)	4	4 (100)	3.5 (3.0–4.3)	5	1 (25)	3	3
8	5	5 (100)	5.0 (4.0–6.0)	6	5 (100)	7 (6.0–7.0)	8	5 (100)	4 (4.0–5.0)	6	5 (100)	4 (4.0–4.0)	5	5 (100)	5 (4.0–5.0)	9	4 (80)	3 (2.8–4.0)	4
9	1	1 (100)	5	5	1 (100)	7	7	1 (100)	7	7	1 (100)	4	4	1 (100)	4	4	0 (0)	–	–
10	3	3 (100)	6 (6.0–6.5)	7	3 (100)	8 (7.5–8.5)	9	3 (100)	7 (6.0–7.0)	7	3 (100)	5 (5.0–6.5)	8	3 (100)	4 (4.0–4.5)	5	1 (33)	4	4
11	5	5 (100)	6 (6.0–6.0)	6	5 (100)	7 (7.0–7.0)	8	5 (100)	5 (4.0–5.0)	5	5 (100)	4 (3.0–4.0)	4	5 (100)	4 (4.0–6.0)	6	3 (60)	3 (2.5–4.0)	5
12	11	11 (100)	6 (5.0–7.0)	8	11 (100)	7 (6.0–8.5)	9	11 (100)	4 (3.5–5.0)	6	11 (100)	5 (4.0–6.0)	7	11 (100)	4 (4.0–4.5)	6	4 (36)	3 (2.8–3.5)	5
13	10	9 (90)	6 (5.0–7.0)	8	10 (100)	7 (6.0–7.0)	11	10 (100)	4.5 (4.0–5.0)	7	8 (80)	5.5 (4.0–6.0)	8	9 (90)	4 (4.0–6.0)	6	3 (30)	2	2
14	10	9 (90)	5 (5.0–6.0)	7	10 (100)	7 (6.0–7.8)	8	10 (100)	5 (4.0–6.0)	12	10 (100)	4 (3.3–4.0)	5	10 (100)	4 (3.3–4.0)	5	3 (30)	3	3
15	14	14 (100)	5.5 (4.0–6.0)	7	14 (100)	7 (5.3–8.0)	12	12 (86)	5.5 (4.0–8.0)	8	13 (93)	4 (4.0–5.0)	7	14 (100)	4 (3.0–4.8)	7	3 (21)	3	3
16	18	17 (94)	5 (5.0–6.0)	11	18 (100)	6 (5.0–8.0)	14	17 (94)	5 (4.0–6.0)	9	16 (89)	5 (3.8–5.0)	9	17 (94)	5 (4.0–6.0)	11	9 (50)	3 (3.0–5.0)	5
17	28	28 (100)	6 (5.0–7.0)	8	28 (100)	7 (6.0–8.0)	11	27 (96)	5 (4.0–6.0)	11	26 (93)	5 (4.0–5.0)	7	27 (96)	5 (4.0–6.0)	9	9 (32)	3 (3.0–4.0)	5

*-* no data available, *IQR* interquartile range, *Max* maximum, *n* number, *yr* years

**Table 2:** Cervical lymph node long-axis size in millimetres in the axial plane by anatomical location

Age *(yr)*	Subjects (*n)*	Level I			Level II			Level III			Level IV			Level V			Level VI		
		*n* (%)	Median (IQR)	Max	*n* (%)	Median (IQR)	Max	*n* (%)	Median (IQR)	Max	*n* (%)	Median (IQR)	Max	*n* (%)	Median (IQR)	Max	*n* (%)	Median (IQR)	Max
1	6	6 (100)	9.5 (9.0–10.8)	13	6 (100)	8.5 (7.3–9.8)	11	6 (100)	6 (5.3–6.8)	11	3 (50)	3 (3.0–4.0)	5	6 (100)	7.5 (7.0–8.0)	9	2 (33)	3.5 (3.3–3.8)	4
2	7	7 (100)	8 (8.0–9.0)	11	7 (100)	11 (8.5–12)	12	6 (86)	8.5 (7.3–9.8)	10	7 (100)	4 (4.0–5.0)	6	7 (100)	7 (5.0–7.0)	8	1 (14)	5	5
3	6	6 (100)	8 (8.0–9.0)	12	6 (100)	11.5 (11.0–12.8)	13	6 (100)	7.5 (7.0–8.0)	8	5 (83)	5 (5.0–6.0)	8	6 (100)	7 (6.3–7.8)	8	3 (50)	4 (3.0–4.5)	5
4	3	3 (100)	10 (7.5–10.0)	10	3 (100)	13 (11.0–14.5)	16	3 (100)	8 (7.5–13.0)	18	3 (100)	3 (3.0–4.0)	5	3 (100)	8 (7.5–8.0)	8	1 (33)	5	5
5	5	5 (100)	10 (10.0–12.0)	16	5 (100)	10 (10.0–13.0)	15	5 (100)	6 (5.0–6.0)	8	5 (100)	5 (5.0–6.0)	9	5 (100)	6 (6.0–7.0)	8	0 (0)	–	–
6	6	5 (83)	10 (8.0–11.0)	14	5 (83)	14 (10.0–16.0)	18	5 (83)	7 (6.0–7.0)	7	5 (83)	6 (6.0–6.0)	7	6 (100)	6 (5.3–6.0)	7	2 (33)	4 (3.5–4.5)	5
7	4	4 (100)	10 (8.3–11.3)	12	4 (100)	11 (10.3–12.5)	17	4 (100)	7 (6.8–8.3)	12	4 (100)	6 (5.3–6.8)	9	4 (100)	5.5 (5.0–7.0)	10	1 (25)	5	5
8	5	5 (100)	7 (7.0–11.0)	15	5 (100)	10 (9.0–11.0)	13	5 (100)	9 (6.0–9.0)	11	5 (100)	6 (6.0–8.0)	9	5 (100)	8 (6.0–9.0)	11	4 (80)	4.5 (3.8–5.5)	7
9	1	1 (100)	8	8	1 (100)	23	23	1 (100)	14	14	1 (100)	10	10	1 (100)	9	9	0 (0)	–	–
10	3	3 (100)	9 (8.5–11.0)	13	3 (100)	14 (12.5–15.0)	16	3 (100)	8 (8.0–9.5)	11	3 (100)	7 (6.5–10.0)	10	3 (100)	7 (6.5–7.0)	7	1 (33)	5	5
11	5	5 (100)	10 (9.0–11.0)	11	5 (100)	12 (7.0–12.0)	15	5 (100)	9 (8.0–9.0)	10	5 (100)	5 (5.0–6.0)	6	5 (100)	8 (8.0–10.0)	10	3 (60)	6 (6.0–7.0)	8
12	11	11 (100)	11 (9.0–12.0)	15	11 (100)	10 (9.0–13.0)	15	11 (100)	6 (6.0–7.0)	10	11 (100)	7 (5.5–8.0)	12	11 (100)	7 (6.0–8.5)	9	4 (36)	4.5 (3.8–5.5)	7
13	10	9 (90)	10 (8.0–12.0)	14	10 (100)	10.5 (10.0–12.0)	18	10 (100)	8 (5.3–8.0)	15	8 (80)	7 (5.5–8.0)	10	9 (90)	7 (6.0–7.0)	10	3 (30)	3 (3.0–3.0)	3
14	10	9 (90)	9 (8.0–10.0)	14	10 (100)	9.5 (8.3–10.8)	12	10 (100)	7 (6.3–8.0)	14	10 (100)	5 (5.0–6.0)	8	10 (100)	7.5 (6.0–9.0)	10	3 (30)	5 (4.5–5.5)	6
15	14	14 (100)	9 (8.0–11.0)	12	14 (100)	10 (8.3–12.8)	24	12 (86)	8 (7.0–9.0)	10	13 (93)	6 (5.0–8.0)	9	14 (100)	7 (6.0–7.8)	12	3 (21)	6 (5.5–7.5)	9
16	18	17 (94)	11 (9.0–14.0)	18	18 (100)	10 (9.0–13.0)	16	17 (94)	9 (7.0–10.0)	13	16 (89)	6 (6.0–7.0)	13	17 (94)	8 (7.0–9.0)	13	9 (50)	6 (5.0–6.0)	11
17	28	28 (100)	12 (9.8–13.0)	17	28 (100)	11 (9.0–13.0)	19	27 (96)	8 (7.0–9.5)	12	26 (93)	7 (5.3–8.0)	11	27 (96)	8 (6.5–9.0)	12	9 (32)	5 (4.0–6.0)	8

*-* no data available, *IQR* interquartile range, *Max* maximum, *n* number, *yr* years

**Table 3:** Cervical lymph node long-axis size in millimetres in the coronal plane by anatomical location

Age *(yr)*	Subjects (*n)*	Level I			Level II			Level III			Level IV			Level V			Level VI		
		*n* (%)	Median (IQR)	Max	*n* (%)	Median (IQR)	Max	*n* (%)	Median (IQR)	Max	*n* (%)	Median (IQR)	Max	*n* (%)	Median (IQR)	Max	*n* (%)	Median (IQR)	Max
1	6	6 (100)	6.5 (6.0–7.0)	8	6 (100)	7.5 (7.0–8.8)	10	6 (100)	6 (6.0–8.3)	11	3 (50)	4 (2.5–4.0)	4	6 (100)	6 (5.0–8.0)	9	2 (33)	2 (2.0–2.0)	2
2	7	7 (100)	7 (6.0–8.5)	9	7 (100)	10 (9.5–13.5)	17	6 (86)	8 (8.0–10.3)	13	7 (100)	5 (3.5–5.0)	7	7 (100)	6 (5.0–6.5)	9	1 (14)	3	3
3	6	6 (100)	8.5 (5.0–9.0)	10	6 (100)	11 (10.0–12.3)	22	6 (100)	7 (4.5–8.9)	13	5 (83)	5 (4.0–6.0)	6	6 (100)	5 (5.0–8.0)	9	3 (50)	2 (1.5–3.5)	5
4	3	3 (100)	7 (7.0–7.5)	8	3 (100)	12 (9.0–14.0)	16	3 (100)	8 (7.0–9.0)	18	3 (100)	4 (3.5–4.5)	5	3 (100)	7.5 (7.3–7.8)	8	1 (33)	4	4
5	5	5 (100)	8 (8.0–11.0)	13	5 (100)	12 (11.0–12.0)	15	5 (100)	8 (7.0–9.0)	18	5 (100)	6 (6.0–7.0)	10	5 (100)	6.5 (5.5–7.3)	8	0 (0)	–	–
6	6	5 (83)	6 (5.0–9.0)	10	5 (83)	14 (10.0–15.0)	18	5 (83)	8 (8.0–9.0)	9	5 (83)	7 (3.0–7.0)	8	5 (83)	8 (7.5–8.5)	10	2 (33)	3 (3.0–3.5)	4
7	4	4 (100)	8 (6.8–9.0)	9	4 (100)	14.5 (11.5–19.0)	25	4 (100)	8 (6.8–10.0)	13	4 (100)	6 (4.5–9.0)	15	4 (100)	6 (5.5–6.5)	7	1 (25)	2	2
8	5	5 (100)	4 (4.0–6.0)	7	5 (100)	11 (10.0–13.0)	15	5 (100)	6 (5.0–11.0)	16	5 (100)	8 (4.0–8.0)	10	5 (100)	8 (7.0–9.0)	9	4 (80)	4 (3.8–4.0)	4
9	1	1 (100)	8	8	1 (100)	23	23	1 (100)	19	19	1 (100)	12	12	1 (100)	9	9	0 (0)	–	–
10	3	3 (100)	12 (7.5–13.5)	15	3 (100)	13 (13.0–17.5)	22	3 (100)	10 (10.0–12.0)	14	3 (100)	7 (7.0–8.5)	10	3 (100)	8 (8.0–8.0)	8	1 (33)	5	5
11	5	5 (100)	8 (8.0–8.0)	8	5 (100)	14 (12.0–14.0)	19	5 (100)	9 (9.0–10.0)	18	5 (100)	7 (4.0–9.0)	10	5 (100)	8 (8.0–10.0)	10	3 (60)	4 (3.5–5.5)	7
12	11	11 (100)	8 (7.0–9.5)	13	11 (100)	12 (11.0–12.5)	22	11 (100)	8 (7.0–10.0)	18	11 (100)	6 (6.0–8.0)	14	11 (100)	8.5 (6.3–9.8)	12	4 (36)	3 (3.0–5.0)	6
13	10	9 (90)	6 (6.0–8.0)	13	10 (100)	13 (8.8–14.8)	22	10 (100)	8 (8.0–8.8)	15	8 (80)	5 (4.0–6.3)	16	9 (90)	8 (6.0–9.3)	13	3 (30)	4 (3.5–4.0)	4
14	10	9 (90)	7 (6.0–9.0)	10	8 (80)	10.5 (10.0–11.3)	14	8 (80)	8 (7.0–10.0)	16	10 (100)	5 (4.0–6.0)	10	10 (100)	8 (7.0–10.0)	15	3 (30)	4 (4.0–4.5)	5
15	14	14 (100)	6.5 (6.0–8.0)	14	13 (93)	12 (10.0–13.0)	28	11 (76)	9 (7.0–11.0)	14	13 (93)	5 (5.0–7.0)	8	13 (93)	8 (7.0–9.0)	12	3 (21)	5 (5.0–5.5)	6
16	18	17 (94)	8 (6.0–10.0)	15	18 (100)	12 (10.0–14.8)	27	17 (94)	9 (7.0–13.0)	18	16 (89)	7 (5.0–9.0)	14	15 (83)	6 (6.0–8.0)	15	9 (50)	5 (3.0–7.0)	10
17	28	27 (96)	8 (5.5–9.5)	16	26 (93)	13 (10.0–14.8)	19	27 (96)	9 (8.0–12.0)	20	26 (93)	7 (4.0–8.0)	15	25 (89)	9 (8.0–11.0)	17	9 (32)	4 (4.0–4.0)	10

*-* no data available, *IQR* interquartile range, *Max* maximum, *n* number, *yr* years

**Fig. 1:**
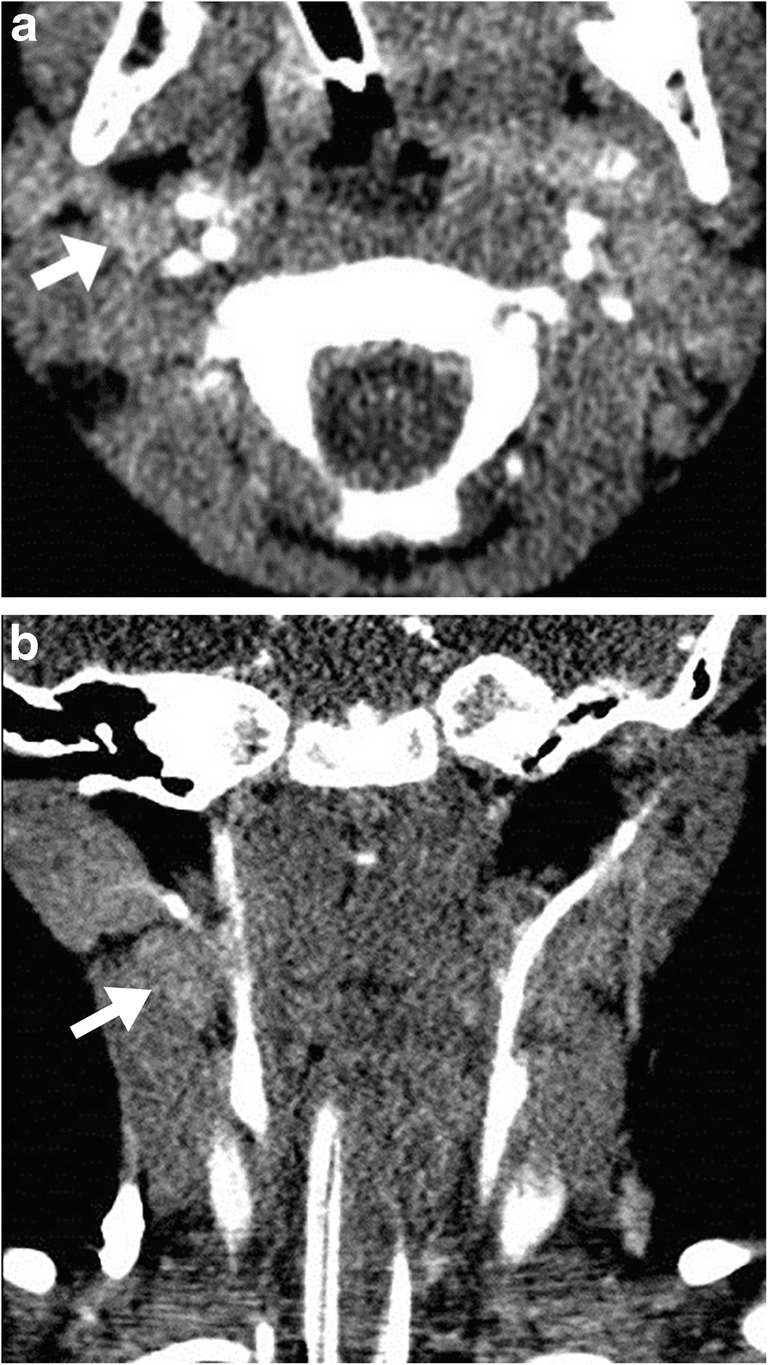
Cervical contrast-enhanced CT images (100 kV, 70 mAs) in a 2-year-old boy. **a** Axial reconstruction shows a Level II lymph node (*arrow*) with a short-axis diameter of 8 mm and a long-axis diameter of 10 mm. **b** Corresponding coronal image shows the same Level II lymph node (*arrow*) with a long-axis diameter of 16 mm. The depicted images provide an example of the vertical alignment of lymph nodes that is often found among cervical lymph node stations

Vertical alignment was noted in 336 of 733 (46%) measured lymph nodes (coronal long-axis diameter exceeded the axial long-axis diameter). This vertical alignment was predominantly found at Levels II through V, where 55% (77/141, Level II), 66% (90/136, Level III), 45% (59/130, Level IV) and 51% (71/139, Level V) of the lymph nodes had a vertical orientation. For Levels I and VI the percentages were 16% (22/138) and 35% (17/49), respectively. Figure 1 shows an example of a vertically orientated lymph node.

**Fig. 2:**
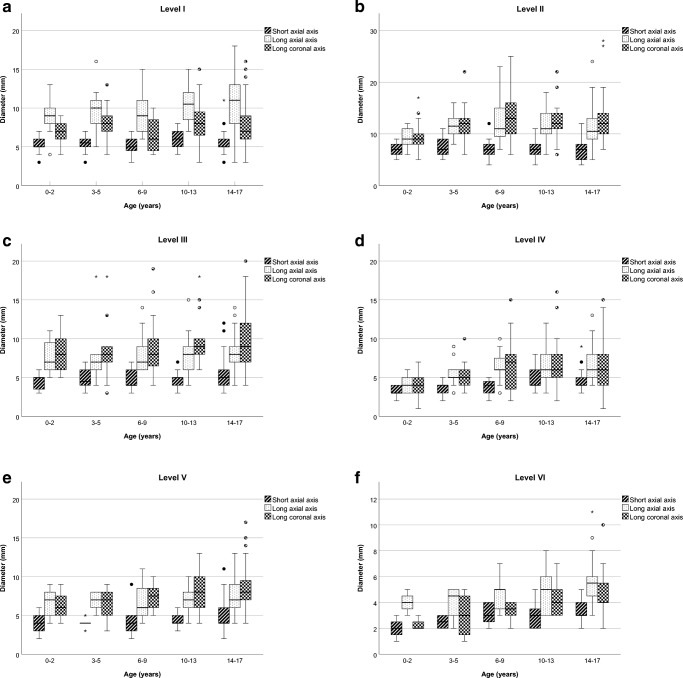
Boxplots (mean, interquartile range, range) for lymph node size at CT by age and axis: (**a**) Level I; (**b**) Level II; (**c**) Level III; (**d**) Level IV; (**e**) Level V; (**f**) Level VI

Table 4 shows the upper limits (mean + 1.96 SD) of the normal range in this paediatric population as calculated per axis and per lymph node level. For the short axial axis diameters, the upper limits of all levels show similarity with current adult guidelines (all levels except for Level II remain less than 10 mm (range 5.63–8.59 mm). For Level II, the upper limit exceeds 10 mm (11.28 mm), with adult guidelines indicating a normal range up to 15 mm. Of all the measured lymph nodes, 11 nodes had a short axial axis higher than 10 mm (1.5% of the measured lymph nodes in 10 children). Those lymph nodes were found in Levels I, II, III and V. When taking into account that for Level II lymph nodes an upper limit of 15 mm is currently used for the short axial axis cut-off point, only 4 lymph nodes would be considered enlarged (0.5%). The long axial axis was above 15 mm in 16 of the measured lymph nodes (2.1%); most of these lymph nodes were found at Level II. For the long axis in the coronal plane, lymph node sizes ranged from 1 mm to 28 mm, and a total of 33 lymph nodes (4.8%) had a diameter exceeding 15 mm. Most of them were found at Levels II and III. Figure 2 shows boxplots for all six lymph node levels. Table 5 shows Pearson correlation coefficients for age and lymph node size. Small to medium significant correlations were seen for Levels IV through VI in all three axes (0.19–0.47, *P*-values <0.05). For Levels I through III, weak correlations were seen for the long axial axis diameter at Level I (0.20, *P=*0.02) and the long coronal axis diameter at Level III (0.18, *P*=0.04).

**Table 4:** Mean and upper limits (mm) of the normal range by axis and lymph node level

Level	Short axial axis		Long axial axis		Long coronal axis	
	Mean	Mean + 1.96 SD	Mean	Mean + 1.96 SD	Mean	Mean + 1.96 SD
Level I	5.44	8.59	9.81	17.10	7.19	14.07
Level II	6.82	11.28	10.67	18.28	11.78	21.90
Level III	4.79	8.39	7.53	12.98	8.80	17.66
Level IV	4.14	7.24	6.01	11.18	5.64	14.12
Level V	4.24	7.76	7.04	11.41	7.52	14.03
Level VI	2.95	5.63	4.82	9.60	3.81	8.83

*SD* standard deviation

**Table 5:** Pearson correlation coefficients for age and lymph node size per level

Node location	Short axial axis	Long axial axis	Long coronal axis
	Coefficient (*P*-value)	Coefficient (*P*-value)	Coefficient (*P*-value)
Level I	NS	0.20 (*P*=0.02)	NS
Level II	NS	NS	NS
Level III	NS	NS	0.18 (*P=*0.04)
Level IV	0.36 (*P*<0.01)	0.39 (*P*<0.01)	0.19 (*P*=0.03)
Level V	0.23 (*P*<0.01)	0.21 (*P=*0.01)	0.36 (*P*<0.01)
Level VI	0.39 (*P*<0.01)	0.33 (*P=*0.02)	0.47 (*P*<0.01)

*NS* not statistically significant

**Fig. 3:**
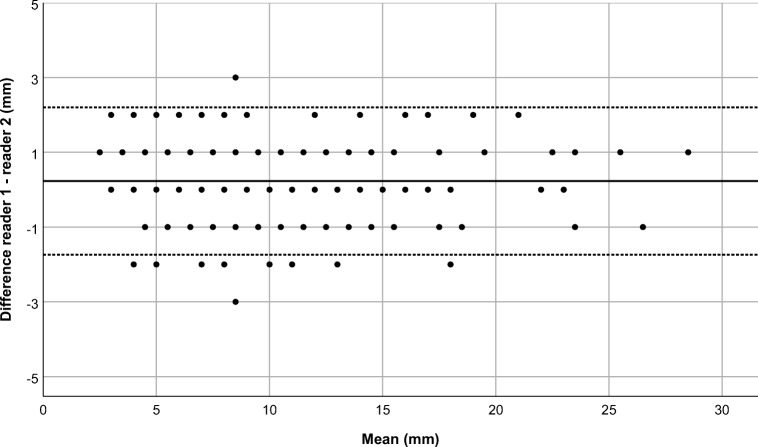
Bland–Altman plot compares lymph node measurements between Reader 1 (A.S.L.) and Reader 2 (S.S.). The difference between the two measurements is plotted against their average. The graph shows measurements from a total of 108 lymph nodes (short- and long-axis diameters in the axial plane and long-axis diameter in the coronal plane) in 20 children. The dotted lines represent the 95% confidence intervals of the average differences (−1.74 mm to 2.20 mm), and the continuous line represents the mean difference (0.23 mm)

Figure 3 shows the Bland–Altman plot for assessing interobserver agreement between readers. The mean difference between measurements was 0.23 mm, and the 95% limits of agreement were −1.74 mm to 2.20 mm.

## Discussion

In this retrospective analysis of cervical CT examinations we provide the prevalence and three axes of diameters for lymph nodes at six cervical levels for children aged 1–17 years. We found a wide variety of maximum lymph node diameters, ranging from 1 mm to 28 mm, in these children. Results show a small to medium correlation between lymph node size and age for Levels IV to VI. This finding is in line with earlier published normal measurements of chest lymph nodes in children [12]. Because the differences in lymph node size with increasing age are small, we believe that age-dependent normal values are not necessary in clinical practise. Additionally, we found that quite often the largest diameter does not correspond to the axial plane in which lymph nodes are most often measured. For cervical lymph nodes it can be concluded that their alignment is often vertical for Levels II, III and V in the majority of nodes. This could be explained by their close proximity to other vertically aligned anatomical structures. Therefore, long- and short-axis diameters measured in the axial plane are likely to be shorter than those measured in the coronal plane. Applying the guidelines for lymph node size to the coronal plane, and possibly the sagittal plane, as well, might therefore lead to different diagnoses of enlarged cervical lymph nodes. This corresponds with an earlier publication in which measurements in all three imaging planes were compared [8].

Current guidelines and literature are inconsistent in terms of cut-off values for normal cervical lymph node sizes. None of them provides guidelines specifically for children. Based on the response evaluation criteria in solid tumours (RECIST), axial short-axis diameters should not exceed 10 mm [13], whereas the Lugano criteria state that axial long-axis diameter can be up to 15 mm without being malignant [6]. However, these guidelines are specifically for malignant diseases and might therefore be inappropriate for distinguishing between normal and abnormal when looking at other causes (e.g., infectious disease). Other authors recommend a cut-off point of 15 mm in the long axis (all imaging planes) for Levels I–III and 10 mm for Levels IV–VI [4] or a short axial axis cut-off of 11 mm for Levels II and III and 10 mm for all other levels [14]. For ultrasound, a short-axis upper limit of 10 mm is generally used, as well [5, 7]. In our clinical practise a cut-off point of 10 mm in short-axis diameter in the axial plane is considered the upper limit for all levels except Level II, where 15 mm is used. With these size limits, four lymph nodes (all in different children) would have been classified as being enlarged at our centre (3% of the included children). Although size is an important criterion in assessing lymph nodes on all imaging modalities, other factors such as shape, borders, internal architecture and enhancement characteristics are of course important, as well [3, 5]. This is especially relevant because interrater variability is to some extent unavoidable when assessing measurements in radiologic images. This variability should be considered, and size alone should not be used to form clinical decisions, but rather other imaging features and the clinical presentation should be taken into account as well, especially when measurements approach the clinically used cut-off points.

There are a couple of limitations to our study that need to be addressed. It would have been preferable to prospectively perform the CT examinations. However, it is not ethically desirable to expose healthy children to ionizing radiation to obtain normal measurements for cervical lymph nodes. Therefore, we used CT scans made in an emergency setting after high-energy trauma to study normal dimensions of lymph nodes. It is our belief that these children form the best available sample of a healthy population of children. Furthermore, it is unlikely that cervical lymph nodes would increase in size minutes to hours after trauma, which was the timeframe in which the scans were made. For some ages the sample size was relatively small, but we were still able to provide normative data for all ages. It should be noted, however, that maximum sizes can be biased by the small sample sizes. Last, the measurements were performed by a doctoral student (S.S.) with limited experience. This limitation was overcome by a an interobserver analysis for a subset of 20 patients with an experienced paediatric radiologist (A.S.L.), and very good interrater reliability was shown.

## Conclusion

When evaluating cervical CT examinations in children, lymph node alignment might not correspond to the axial plane. In the majority of children, regardless of age, the axial short-axis diameter of lymph nodes is <15 mm for Level II lymph nodes and <10 mm for lymph nodes at all other cervical levels.
